# Effects of Pressure-Induced Density Changes in the Thermal Energy Absorbed by a Micro-Encapsulated Phase-Change Material

**DOI:** 10.3390/molecules24071254

**Published:** 2019-03-30

**Authors:** Ernesto M. Hernández-Cooper, José A. Otero

**Affiliations:** Departamento de Ciencias, Escuela de Ingeniería y Ciencias, Tecnológico de Monterrey Campus Estado de México, Atizapán de Zaragoza, Estado de México 52900, México; emcooper@tec.mx

**Keywords:** phase-change material, micro-encapsulated, thermal energy absorbed, sensible heat, latent heat

## Abstract

Density changes produced by pressure increments during melting of a spherically confined phase-change material have an impact on the thermal energy absorbed by the heat storage unit. Several authors have assumed incompressible phases to estimate the volume change of the phase-change material and the thermal balance at the liquid–solid interface. This assumption simplifies the problem but neglects the contribution of density changes to the thermal energy absorbed. In this work, a thermal balance at the interface that depends on the rate of change of the densities and on the shape of the container is found by imposing total mass conservation. The rigidity of the container is tuned through the coupling constant of an array of springs surrounding the phase-change material. This way, the behavior of the system can be probed from the isobaric to the isochoric regimes. The sensible and latent heat absorbed during the melting process are obtained by solving the proposed model through numerical and semi-analytical methods. Comparing the predictions obtained through our model, it is found that even for moderate pressures, the absorbed thermal energy predicted by other authors can be significantly overestimated.

## 1. Introduction

Phase-change materials (PCMs) have provided an extensive line of research due to their appealing applications in areas related to renewable energy systems for the reduction of fossil fuel consumption. These materials are used to provide thermal comfort in homes and buildings by exploiting the isothermal nature of first-order phase transitions [1,2,3,4]. Thermal isolation is provided by using the latent heat of PCMs to absorb thermal energy during the day and releasing this energy during the night hours [5,6]. PCMs may be encapsulated in micro-spheres to enhance thermal transport [7], and may be mixed with the concrete wall in volumetric fractions that do not compromise the mechanical resistance of constructions [8,9]. High-temperature phase-change materials (HTPCMs) constitute another type of application, where PCMs with high fusion temperatures are used to store thermal energy in concentrating solar power (CSP) plants for thermoelectric energy generation [10]. The micro-encapsulated materials are used as a part of the thermal energy storage (TES) unit, which feeds the thermoelectric plant through a heat-transfer fluid (HTF) during those hours without sunlight [11]. Enhancement of thermal conductivities to reduce the charging and discharging times on HTPCMs has also been addressed by studying composite materials [12,13,14]. Finally, studies on other enhancement techniques to reduce the duration of the charging–discharging cycles for domestic heat recovery applications have been addressed as well [15].

When thermal performance of buildings is achieved by mixing the micro-encapsulated material within the concrete matrix, the PCM cannot expand freely during the melting process. PCMs are expected to expand while melting, since most of these materials have lower densities in their liquid state than in the solid state [15,16]. Volumetric expansion of HTPCMs is also constrained when encapsulated in metallic shells, since the elastic modulus of the shell is much higher than the bulk modulus of the PCM in its liquid and solid states [17]. The melting process of the PCM in all these types of applications takes place near the isochoric regime (constant volume). Thermo-mechanical models have been developed for encapsulated HTPCMs in spherical configurations [18,19,20,21]. These proposals constitute a first approach for explaining the behavior of PCMs when encapsulated by different materials. Other authors [22,23], study the one-dimensional liquid–solid phase transition, by coupling the PCM to a linear spring and assuming incompressible phases. This assumption neglects the effects of density variations on the behavior of the phase transition. Other authors avoid the effect of density changes during the phase transition by introducing an air void [21]. More recently, a proposal for incorporating density changes during the melting process has been developed for a one-dimensional plane problem [24]. Large differences between the time evolution of the thermodynamic variables in [18,19,20,24] are predicted at high pressures.

In this work, we study the effects of pressure-induced density changes, on the thermal energy absorbed by a micro-encapsulated HTPCM. As mentioned earlier, these type of PCMs are used as part of the heat storage units for thermoelectric generation during periods without sunlight. Therefore, the thermal storage capability of the PCM has a direct impact on the efficiency of CSP plants. The thermo-mechanical model presented in this paper has been elaborated through the key idea of total and local mass conservation in a spherically confined PCM. Our proposal leads to an estimation of the sensible heat, latent heat, and total heat absorbed by the PCM that presents significant differences when compared to the predictions of other authors [18,19,20,22,23]. Through mass conservation, an extra term that depends on the shape of the container and the rate of density changes, will be shown to appear on the thermal balance at the interface. To the authors knowledge, this term has been overlooked or neglected by other authors [18,19,20,22,23]. We will show that depending on the mechanical properties of the container, this term represents a small perturbation, or it may have a significant contribution to the thermal energy absorbed by the PCM.

First, to validate the proposed solutions, an isochoric limit will be presented. In this limit, the container is completely rigid and through mass conservation, exact analytical expressions for the dynamic and thermodynamic variables will be found. An implicit finite difference method (FDM) and a refined heat balance integral method (RHBIM) will be developed to solve the proposed model. The numerical and semi-analytical solutions will be validated through the isochoric limit, by probing the phase transition from the isobaric (constant pressure) to the isochoric regimes. The thermo-mechanical models proposed by other authors [18,19,20,22,23] are well behaved close to the isobaric regime; however, it will be shown that these models are unable to reproduce the isochoric limit. Finally, the contributions from density changes to the thermal energy absorbed, will be determined by comparing the proposed solutions with the predictions from other authors. It will be shown that sensible heat must be conceived in four stages, where mass conservation plays a key role. For the chosen PCM, examples will be presented, where the absorbed thermal energy is overestimated by other authors. The difference in the absorbed thermal energy according to the numerical and semi-analytical solutions to both models is observed to grow significantly with the rigidity of the container.

## 2. Results and Discussion

### 2.1. Description of the Physical System

**Figure 1:**
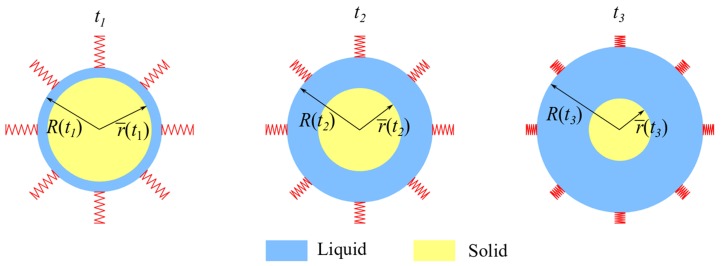
Schematic representation of the time evolution of the melting process. As time evolves, the radius of the solid phase $\bar{r}\left(t\right)$ moves inwards and the system size R(t) increases.

The system under study consists of an encapsulated PCM in a spherical shell of radius R(t) at any time *t*, where liquid and solid phases coexist, so that the liquid–solid interface at any time *t* is located at $r=\bar{r}\left(t\right)$. The boundary of the spherical configuration located at r=R(t) is attached to a set of springs as shown in Figure 1. The system is subjected to the following boundary conditions

(1) $$\begin{array}{ccc} T_{\ell }\left(r,t\right){|}_{r=R(t)} & = & T_{H},\\ \lambda_{s}\;\frac{\partial \;T_{s}\left(r,t\right)}{\partial \;r}{|}_{r=0} & = & 0,\\ T_{\ell }\left(r,t\right){|}_{r=\bar{r}\left(t\right)} & = & T_{s}\left(r,t\right){|}_{x=\bar{r}\left(t\right)}=T_{f}\left(t\right), \end{array}$$

where $\lambda_{s}$ is the thermal conductivity of the solid phase and $T_{\ell }\left(r,t\right)$$\left(T_{s}\left(r,t\right)\right)$ is the temperature distribution in the liquid(solid) phase at any instant *t*. The temperature $T_{H}$ at the surface of the PCM is always above the fusion temperature $T_{f}\left(t\right)$. Then, the chosen boundary conditions will produce melting of the PCM so that $\bar{r}\left(t\right)$ moves inwards as shown in Figure 1. Within the temperature range that will be considered, thermal expansion effects can be neglected. This assumption implies that pressure will be distributed uniformly along the PCM. The stiffness of the spring array shown in Figure 1 will be tuned through the spring constant $k_{s}$ to study the behavior of the system.

### 2.2. Energy-Mass Balance at the Interface and Heat-Transfer Mechanism

In this part of the present section, the energy-mass balance equation (EMB) at the interface and the proposed model for heat transfer, will be presented for a melting process when the PCM is under mechanical stress. In [18,19,20], the solid phase is assumed to be incompressible. Then, the effects of density changes in the solid, caused by the pressure increment within the PCM can be neglected. In this work, the assumption of incompressible phases [22,23] will be relaxed by coupling the mass conservation of the liquid–solid system with the thermal balance at the interface. First, the melted mass of solid $\Delta M_{s}$ over a small time interval Δt, can be obtained by realizing that $\Delta M_{s}$ is transformed into liquid. Additionally, if the total mass of the system is conserved:

(2) $$\Delta M_{s}=M_{\ell }\left(t+\Delta t\right)-M_{\ell }\left(t\right),$$

where $M_{\ell }\left(t+\Delta t\right)$ is the mass of liquid at t+Δt, after the melted solid $\Delta M_{s}\left(t\right)$ is added to the initial mass of liquid $M_{\ell }\left(t\right)$. Therefore, the thermal energy needed to melt the mass of solid $\Delta M_{s}$ within a small time interval Δt is given by

(3) $$\begin{array}{ccc} L_{f}\left(t\right)\;\frac{dM_{\ell }\left(t\right)}{dt} & = & 4\;\pi \;L_{f}\left(t\right)\;{\bar{r}}^{2}\left(t\right)\;\sigma_{\ell },\;\;\;\mathrm{where}\\ \sigma_{\ell } & = & \rho_{\ell }\left(t\right)\;\left(\frac{R^{2}\left(t\right)}{{\bar{r}}^{2}\left(t\right)}\frac{dR(t)}{dt}-\frac{d\bar{r}\left(t\right)}{dt}\right)+\frac{1}{3}\;\frac{d\rho_{\ell }\left(t\right)}{dt}\left(\frac{R^{3}\left(t\right)}{{\bar{r}}^{2}\left(t\right)}-\bar{r}\left(t\right)\right). \end{array}$$

Here $M_{\ell }\left(t\right)=4\;\pi \;\rho_{\ell }\left(t\right)\left(R^{3}\left(t\right)-{\bar{r}}^{3}\left(t\right)\right)/3$ has been used. The right-hand side of the last equation is equal to the net heat flux at $r=\bar{r}\left(t\right)$. Then, the following EMB equation at the interface is obtained:

(4) $$L_{f}\left(t\right)\;\sigma_{\ell }=\lambda_{\ell }\frac{\partial \;T_{\ell }\left(r,t\right)}{\partial \;r}{|}_{r=\bar{r}\left(t\right)}-\lambda_{s}\frac{\partial \;T_{s}\left(r,t\right)}{\partial \;r}{|}_{r=\bar{r}\left(t\right)}\;,$$

where $\lambda_{\ell }$($\lambda_{s}$) is the thermal conductivity of the liquid(solid) phase.

Imposing mass conservation to the entire PCM, another equation for the densities and dynamical variables of motion can be obtained as follows

(5) $$\begin{array}{ccc} \frac{dM_{\mathrm{PCM}}\left(t\right)}{dt} & = & {\bar{r}}^{2}\left(t\right)\left(\sigma_{s}+\sigma_{\ell }\right)=0,\;\;\;\mathrm{where}\\ \sigma_{s} & = & \rho_{s}\left(t\right)\frac{d\bar{r}\left(t\right)}{dt}+\frac{1}{3}\frac{d\rho_{s}\left(t\right)}{dt}\bar{r}\left(t\right). \end{array}$$

Using this expression, Equation (4) can be written in terms of the density of the solid as

(6) $$L_{f}\left(t\right)\;\sigma_{s}=-\lambda_{\ell }\frac{\partial \;T_{\ell }\left(r,t\right)}{\partial \;r}{|}_{r=\bar{r}\left(t\right)}+\lambda_{s}\frac{\partial \;T_{s}\left(r,t\right)}{\partial \;r}{|}_{r=\bar{r}\left(t\right)}.$$

This equation is equivalent to the EMB Equation (4) as long as mass conservation is imposed. The pressure increment ΔP within the liquid is equal to the increment in pressure within the solid phase. Using the bulk modulus of each phase [24], a relation between the densities in both media is given by:

(7) $$B_{\ell }\left(\frac{\rho_{\ell }\left(t+\Delta t\right)-\rho_{\ell }\left(t\right)}{\rho_{\ell }\left(t+\Delta t\right)}\right)=B_{s}\left(\frac{\rho_{s}\left(t+\Delta t\right)-\rho_{s}\left(t\right)}{\rho_{s}\left(t+\Delta t\right)}\right),$$

where $B_{\ell }$($B_{s}$) is the bulk modulus of the liquid(solid) phase. The pressure increment at any time *t* can be obtained from the liquid or solid deformation given by the last equation, as follows

(8) $$\Delta P=B_{\ell }\left(\frac{\rho_{\ell }\left(t\right)-\rho_{\ell }\left(0\right)}{\rho_{\ell }\left(t\right)}\right)\;\;\;\mathrm{or}\;\;\;\Delta P=B_{s}\left(\frac{\rho_{s}\left(t\right)-\rho_{s}\left(0\right)}{\rho_{s}\left(t\right)}\right).$$

The deformation that each medium experiences during the phase transition is coupled to the elastic properties of the spring array surrounding the spherical configuration, which is assumed to obey Hooke’s law. Then, the deformation of the liquid phase coupled to the elastic constant of the springs is given by:

(9) $$B_{\ell }\left(\frac{\rho_{\ell }\left(t+\Delta t\right)-\rho_{\ell }\left(t\right)}{\rho_{\ell }\left(t+\Delta t\right)}\right)={\tilde{k}}_{s}\;\frac{R(t+\Delta t)-R(t)}{R_{0}},$$

where ${\tilde{k}}_{s}=k_{s}\;N_{s}\;R_{0}$ which will be expressed in multiples of $B_{\ell }$, and $R_{0}$ is the initial radius of the PCM. $N_{s}$ is the concentration of springs over the surface of the system. Changing this parameter, will allow the probing of the behavior of the phase transition for different pressure regimes. Equations (4), (6), (7) and (9) or alternatively, Equations (6), (7) and (9) constitute a set of nonlinear equations for the dynamical variables $\bar{r}\left(t\right)$ and R(t), and the densities of each phase.

The liquid–solid saturation line is obtained through a second order approximation in ΔP and $\Delta T_{f}$ of the free energy change as described in [18]. In this approximation the fusion temperature is given by:

(10) $$\begin{array}{ccc} \Delta T_{f} & = & \frac{-b+\sqrt{b^{2}-4\;a\;c}}{2\;a}\;\;\;\mathrm{with}\\ a & = & \frac{1}{2\;T_{f}\left(0\right)}\left(C_{\ell }-C_{s}\right),\\ b & = & \frac{L_{f}\left(0\right)}{T_{f}\left(0\right)}-\left(\frac{\alpha_{\ell }}{\rho_{\ell }\left(0\right)}-\frac{\alpha_{s}}{\rho_{s}\left(0\right)}\right)\;\Delta P,\\ c & = & \frac{1}{2}\left(\frac{K_{\ell }}{\rho_{\ell }\left(0\right)}-\frac{K_{s}}{\rho_{s}\left(0\right)}\right)\;\Delta P^{2}-\left(\frac{1}{\rho_{\ell }\left(0\right)}-\frac{1}{\rho_{s}\left(0\right)}\right)\;\Delta P, \end{array}$$

where $\Delta T_{f}=T_{f}\left(t\right)-T_{f}\left(0\right)$. $K_{\ell }$($K_{s}$) is the compressibility of the liquid(solid) phase, which can be obtained as the inverse of the bulk modulus; $\alpha_{\ell }$($\alpha_{s}$) is the liquid(solid) thermal expansion coefficient and $C_{\ell }$($C_{s}$) is the specific heat capacity of the liquid(solid) phase. Then, the latent heat of fusion can be obtained as follows [18]

(11) $$L_{f}\left(t\right)=L_{f}\left(0\right)\frac{T_{f}\left(t\right)}{T_{f}\left(0\right)}+\left(C_{\ell }-C_{s}\right)\;\Delta T_{f}\frac{T_{f}\left(t\right)}{T_{f}\left(0\right)}-T_{f}\left(t\right)\;\Delta P\;\left(\frac{\alpha_{\ell }}{\rho_{\ell }\left(0\right)}-\frac{\alpha_{s}}{\rho_{s}\left(0\right)}\right).$$

The heat equation for any phase i=liquid(solid) that is consistent with local mass conservation [24,25] is given in spherical coordinates as

(12) $$\rho_{i}\left(t\right)\;C_{i}\;\frac{\partial \;T_{i}\left(r,t\right)}{\partial \;t}=\lambda_{i}\;\frac{1}{r^{2}}\frac{\partial \;}{\partial \;r}\left(r^{2}\frac{\partial \;T_{i}\left(r,t\right)}{\partial \;r}\right).$$

Finally, the heat equation presented in [18,19,20], and given by:

(13) $$\;C_{i}\;\frac{\partial \;\left(\rho_{i}\left(t\right)\;T_{i}\left(r,t\right)\right)}{\partial \;t}=\lambda_{i}\;\frac{1}{r^{2}}\frac{\partial \;}{\partial \;r}\left(r^{2}\frac{\partial \;T_{i}\left(r,t\right)}{\partial \;r}\right),$$

will be used to describe the heat transfer within each phase according to the theory presented in [18].

### 2.3. Absorbed Sensible and Latent Heat

Sensible heat is absorbed through four different stages that will be described briefly. Mass conservation also plays a key role when considering the thermal energy absorbed by the PCM. This process has been studied for isobaric phase transitions [25]; however, the mechanism by which sensible heat is absorbed in this case is completely different, since the liquid–solid saturation curve must be considered in the process. The heat that enters the PCM between *t* and t+Δt is absorbed as sensible heat in the following stages:

(a) The mass of solid that will not experience a phase transition between *t* and t+Δt absorbs part of the heat by changing its temperature from $T_{s}\left(r,t\right)$ to $T_{s}\left(r,t+\Delta t\right)$,
(b) The mass of solid $\Delta M_{s}$ absorbs heat before changing to its liquid form by raising its temperature from $T_{s}\left(r,t\right)$ to the fusion temperature $T_{f}\left(t\right)$,
(c) Once transformed to its liquid form, $\Delta M_{s}$ absorbs sensible heat by changing its temperature from $T_{f}\left(t+\Delta t\right)$ to $T_{\ell }\left(r,t+\Delta t\right)$. At this point, the fusion temperature has changed according to Equation (10), since the inner pressure increases after the phase transition,
(d) The original mass of liquid at time *t* absorbs heat by increasing its temperature from $T_{\ell }\left(r,t\right)$ to $T_{\ell }\left(r,t+\Delta t\right)$.

For each of the processes described above, the absorbed sensible heat will be obtained through the internal energy change. During the first stage, the mass of solid that does not experience a phase transition within the time interval Δt is equal to $M_{s}\left(t+\Delta t\right)=M_{s}\left(t\right)-\Delta M_{s}$. The internal energy change experienced by this mass is given by:

(14) $$\Delta U_{a}=4\;\pi \;C_{s}\;\left(\rho_{s}\left(t+\Delta t\right)\;\int_{0}^{\bar{r}\left(t+\Delta t\right)}T_{s}\left(r,t+\Delta t\right)\;r^{2}\;dr-\rho_{s}\left(t\right)\;\int_{0}^{r_{s}}T_{s}\left(r,t\right)\;r^{2}\;dr\right),$$

where $r_{s}$ is the radius at time *t* of the solid sphere with mass $M_{s}\left(t+\Delta t\right)$ and the interface position $\bar{r}\left(t+\Delta t\right)$ is the radius of this same mass of solid at time t+Δt. Then, applying mass conservation to $M_{s}\left(t+\Delta t\right)$ between *t* and t+Δt, the value of $r_{s}$ can be obtained as

(15) $$r_{s}=\bar{r}\left(t+\Delta t\right)\;{\left(\frac{\rho_{s}\left(t+\Delta t\right)}{\rho_{s}\left(t\right)}\right)}^{1/3}.$$

During the second stage, the mass of solid $\Delta M_{s}$ is distributed along a spherical shell that occupies a volume equal to $\Delta V_{s}=4\;\pi \;\left({\bar{r}}^{3}\left(t\right)-r_{s}^{3}\right)/3$, before the phase transition. Therefore, the internal energy change experienced by this mass of solid is given by

(16) $$\Delta U_{b}=C_{s}\;\Delta M_{s}\;T_{f}\left(t\right)-4\;\pi \;C_{s}\;\rho_{s}\left(t\right)\int_{r_{s}}^{\bar{r}\left(t\right)}T_{s}\left(r,t\right)\;r^{2}\;dr.$$

After the phase transition, the pressure has changed and the fusion temperature has increased to $T_{f}\left(t+\Delta t\right)$. Therefore, the internal energy change experienced by the melted mass $\Delta M_{s}$ is given by:

(17) $$\Delta U_{c}=4\;\pi \;C_{\ell }\;\rho_{\ell }\left(t+\Delta t\right)\int_{\bar{r}\left(t+\Delta t\right)}^{r_{\ell }}T_{\ell }\left(r,t+\Delta t\right)\;r^{2}\;dr-C_{\ell }\;\Delta M_{s}\;T_{f}\left(t+\Delta t\right),$$

where $r_{\ell }-\bar{r}\left(t+\Delta t\right)$ is the thickness of a spherical shell with mass $\Delta M_{s}$, in its liquid form. The melted mass $\Delta M_{s}=M_{s}\left(t\right)-M_{s}\left(t+\Delta t\right)$ can be found in terms of $\rho_{s}\left(t\right)$ and $\rho_{s}\left(t+\Delta t\right)$. Additionally, using the volume of the liquid shell associated with this mass, the value of $r_{\ell }$ can be obtained as follows

(18) $$r_{\ell }={\left(\frac{\rho_{s}\left(t\right)}{\rho_{\ell }\left(t+\Delta t\right)}\;{\bar{r}}^{3}\left(t\right)-\left(\frac{\rho_{s}\left(t+\Delta t\right)}{\rho_{\ell }\left(t+\Delta t\right)}-1\right)\;{\bar{r}}^{3}\left(t+\Delta t\right)\right)}^{1/3}.$$

Finally, the mass of liquid present in the system at time *t*, experiences the following change of internal energy

(19) $$\Delta U_{d}=4\;\pi \;C_{\ell }\left(\rho_{\ell }\left(t+\;\Delta t\right)\;\int_{r_{\ell }}^{R(t+\Delta t)}T_{\ell }\left(r,t+\Delta t\right)\;r^{2}\;dr-\rho_{\ell }\left(t\right)\;\int_{\bar{r}\left(t\right)}^{R(t)}T_{\ell }\left(r,t\right)\;r^{2}\;dr\right).$$

Adding the contributions from Equations (14), (16), (17) and (19), the absorbed sensible heat is obtained. The energy absorbed as latent heat within the time interval Δt is given by

(20) $$\Delta Q_{f}=L_{f}\left(t\right)\;\Delta M_{s}.$$

The total heat absorbed by the PCM is obtained by adding the results from Equations (14), (16), (17), (19) and (20) as follows

(21) $$\Delta Q=\Delta U_{a}+\Delta U_{b}+\Delta U_{c}+\Delta U_{d}+\Delta Q_{f}.$$

### 2.4. Initial Conditions

The results obtained from the numerical and semi-analytical methods applied to the proposed model, will be presented in two parts. First, we will obtain the pressure, density, and liquid–solid saturation line for the nitrate salt considered [18], in a wide range of pressures where the approximation established by Equations (10) and (11) is valid. Initially, the spherical configuration will be almost in its solid state with a very thin liquid layer surrounding the solid, and the melting process will be studied until practically all the solid has melted. Next, we will present the results for the sensible heat, latent heat, and total energy absorbed by the salt, for different values of the spring constant. The absorbed thermal energy obtained with the proposed model, will be compared with the results predicted by the thermo-mechanical model presented in [18].

All the results that will be presented in this section correspond to a PCM confined in a spherical configuration with an initial radius of R(0)=1.0mm. The solid phase is centered at the origin and has an initial radius of $\bar{r}\left(0\right)=0.99\;\mathrm{mm}$. The solid is surrounded by a thin liquid layer of $R\left(0\right)-\bar{r}\left(0\right)=0.001\;\mathrm{mm}$ of thickness. Initially, the inner pressure of the system is P(0)=1atm. The temperature at the surface of the PCM is fixed at $T_{H}=550\;K$. The net heat flux vanishes at the origin of the configuration according to Equation (1), and initially the temperature at r=0 is equal to $T_{C}=420\;K$. Large values of the bulk modulus of the solid phase $B_{s}$ will be assumed, to compare the predicted results from the proposed model, with the results from the model introduced in [18]. The thermodynamic variables at 1atm of pressure are given in Table 1. Pressure-induced changes in the thermal conductivities, specific heat capacities, bulk modulus, and thermal expansion coefficients are neglected.

### 2.5. Numerical and Semi-Analytical Results: Isobaric and Isochoric Regimes

The authors in [18] assume an incompressible solid. In this approximation, the proposed thermal balance at the interface is given by [18]:

(22) $$Lf\left(t\right)\rho_{s}\left(t\right)\frac{d\bar{r}\left(t\right)}{dt}=-\lambda_{\ell }\frac{\partial \;T_{\ell }\left(r,t\right)}{\partial \;r}{|}_{r=\bar{r}\left(t\right)}+\lambda_{s}\frac{\partial \;T_{s}\left(r,t\right)}{\partial \;r}{|}_{r=\bar{r}\left(t\right)}.$$

which neglects the form factor given by Equation (6). The model presented in [18], estimates the volume change of the PCM in terms of the volumetric fraction of melted solid as follows:

(23) $$\Delta V=V_{\mathrm{PCM}}\left(t\right)-V_{\mathrm{PCM}}\left(0\right)=V_{s}\left(0\right)\left(\frac{\rho_{s}\left(0\right)}{\rho_{\ell }\left(0\right)}-1\right)\;f_{v},$$

where $f_{v}=\left(V_{s}\left(0\right)-V_{s}\left(t\right)\right)/V_{s}\left(0\right)$ is the volumetric fraction of melted solid. In this equation, the changes in the liquid and solid densities are neglected; therefore, it will only be valid when the transition takes place for ${\tilde{k}}_{s}\ll \;B_{\ell }$, which is close to the isobaric regime. Then, the thermal balance at the interface given by Equation (22), total volume expansion described by Equation (23) and Equations (9)–(11) and (13) represent the thermo-mechanical model proposed in [18,19,20].

The solutions to the model introduced in [18] and the proposed model in this work, will be compared for several values of ${\tilde{k}}_{s}$. Although the model proposed in this work does not need to assume an incompressible solid, $B_{s}\gg \;B_{\ell }$ and $B_{s}\gg \;{\tilde{k}}_{s}$ will be assumed, to compare our results with the model proposed in [18]. For each value of the spring constant, the melting process will be studied until the mass fraction of melted solid is $f_{s}=\left(M_{s}\left(0\right)-M_{s}\left(t_{\max}\right)\right)/M_{s}\left(0\right)=0.999$. The density of the liquid, the inner pressure, the fusion temperature and latent heat of fusion will be obtained from both models at full melting fractions and for several values of ${\tilde{k}}_{s}$. An isochoric limit will be derived to establish the validity of the solutions to each model. In this limit ${\tilde{k}}_{s}\gg \;B_{\ell }$, and the volume of the spherical configuration does not change during the phase transition. At full melting, it is possible to obtain the density of the liquid for an isochoric transition by using mass conservation. When the total mass is conserved, the initial mass of the PCM $M_{\mathrm{PCM}}\left(0\right)$ is equal to the total mass at full melting $M_{\mathrm{PCM}}\left(t_{\max}\right)$. Additionally, when the solid melts at constant volume, $R\left(0\right)=R\left(t_{\max}\right)$. Then, the maximum possible value for the density of the liquid $\rho_{\ell }^{*}=\rho_{\ell }\left(t_{\max}\right)$ in this limit is given by

(24) $$\rho_{\ell }^{*}=\rho_{\ell }\left(0\right)\left(1+\delta_{r}\;\delta_{\rho }\right),\;\;\;\mathrm{where}\;\;\;\delta_{r}={\left(\frac{\bar{r}\left(0\right)}{R(0)}\right)}^{3}\;\;\mathrm{and}\;\;\delta_{\rho }=\left(\frac{\rho_{s}\left(0\right)}{\rho_{\ell }\left(0\right)}-1\right).$$

The inner pressure in this limit is obtained by substituting Equation (24) in Equation (8); therefore, the isochoric limit for the inner pressure is given by

(25) $$P^{*}=P\left(0\right)+B_{\ell }\;\frac{\delta_{r}\;\delta_{\rho }}{1+\delta_{r}\;\delta_{\rho }}$$

**Figure 2:**
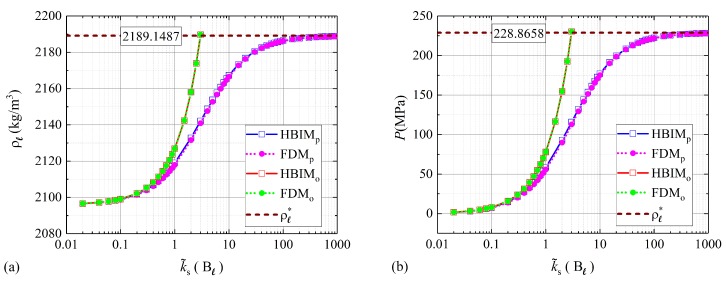
(**a**) Liquid density and (**b**) inner pressure at $f_{s}=0.999$. Numerical and semi-analytical solutions to the proposed model are shown in pink and blue symbols. The solutions to the model described in [18] are shown in green and red symbols. The dashed line corresponds to the asymptotic values for the density of the liquid $\rho_{\ell }^{*}$ and inner pressure $P^{*}$.

The isochoric values at full melting fractions for the fusion temperature and latent heat of fusion are obtained by substituting $\Delta P^{*}=P^{*}-P\left(0\right)$ in Equations (10) and (11). Figure 2, shows the results obtained for the liquid density and inner pressure at mass fractions $f_{s}=0.999$ of melted solid and for a wide range of spring constant values ${\tilde{k}}_{s}=\left[0.02\;B_{\ell },1000\;B_{\ell }\right]$. This range of ${\tilde{k}}_{s}$ has been chosen to probe the phase transition from the isobaric to the isochoric regimes and to check the validity of the proposed model through the isochoric limits given by Equations (24) and (25).

The results shown in Figure 2 confirm that the model proposed in this work is consistent with the isochoric limit and is well behaved near this regime. Since Equations (22) and (23) are valid for weak couplings or small values of ${\tilde{k}}_{s}$, where density changes can be neglected, the approximation presented in [18] is observed to be in good agreement with the model proposed in this work. In this regime, the form factor in Equation (4) and density variations are just a small perturbation to the solution of the proposed model. However, as illustrated in Figure 2, when the spring constant ${\tilde{k}}_{s}$ is increased and the phase transition deviates from the isobaric regime, the solutions to the model proposed in [18] start to diverge from the isochoric limit given by Equations (24) and (25).

**Figure 3:**
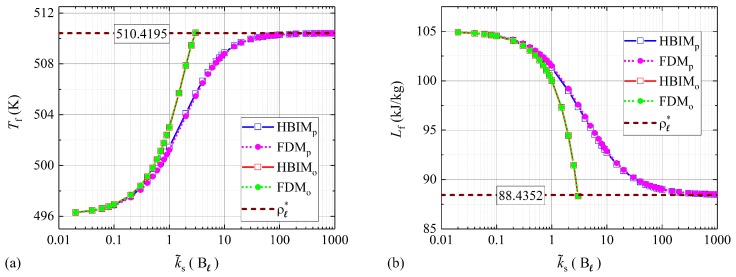
(**a**) Latent heat of fusion and (**b**) fusion temperature for $f_{s}=0.999$. Numerical and semi-analytical solutions to the proposed model are shown in pink and blue symbols, and the solutions to the model presented in [18] are shown in green and red symbols. The isochoric limits for $T_{f}^{*}$ and $L_{f}^{*}$ are shown with a dashed line.

In Figure 3, the results for $L_{f}$ and $T_{f}$ at melted solid fractions of $f_{s}=0.999$ are shown. The temperature of fusion and latent heat of fusion are very important thermodynamic variables when estimating the total heat absorbed by the PCM during the melting process. Therefore, the solutions obtained from the proposed model for these thermodynamic variables are also validated through the isochoric limit found by substitution of $\Delta P^{*}=P^{*}-P\left(0\right)$ in Equations (10) and (11). As illustrated in Figure 3, the numerical and semi-analytical solutions to the model proposed in this work, also show a good agreement with the liquid–solid saturation point in this limit. Also as expected, the solutions to the thermo-mechanical model presented in [18] are well behaved in the isobaric regime but tend to diverge from the isochoric limit as the spring constant ${\tilde{k}}_{s}$ is increased.

Equation (23) is a statement of mass conservation for isobaric transitions, and it can be obtained from Equation (6), when density variations are neglected. The interpretation of the large differences between the two models for P≥50MPa as observed in Figure 2 can be inferred from the principle of mass conservation. Equation (6) can be expressed in terms of the total volume change experienced by the PCM during the melting process. Assuming that $\rho_{s}$ does not change with pressure increments and keeping only first-order terms in $\Delta \rho_{\ell }=\rho_{\ell }\left(t\right)-\rho_{\ell }\left(0\right)$, the volume change according to Equation (6) is given by

(26) $$\Delta V=V_{s}\left(0\right)\left[\left(\frac{\rho_{s}\left(0\right)}{\rho_{\ell }\left(0\right)}-1\right)-\left(\frac{\rho_{s}\left(0\right)}{\rho_{\ell }\left(0\right)}\frac{\Delta \rho_{\ell }}{\rho_{\ell }\left(0\right)}\right)\right]\;f_{v}.$$

**Figure 4:**
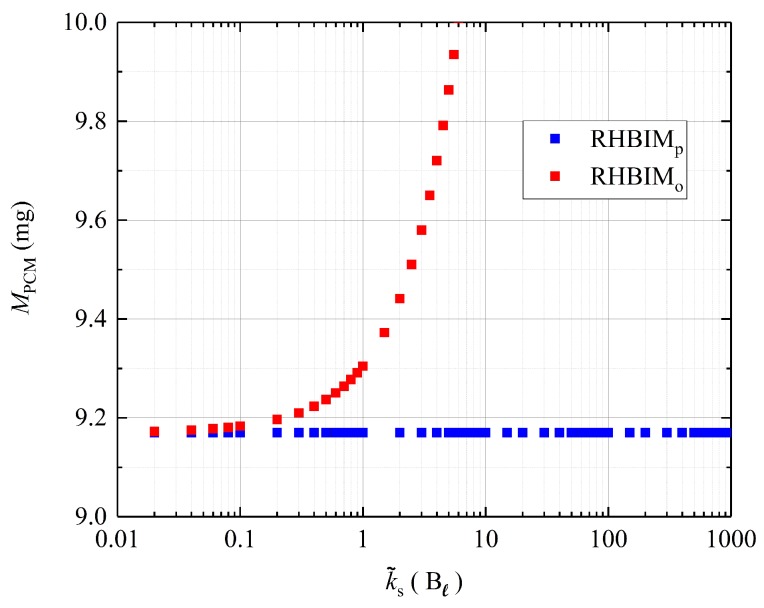
Total mass of the salt for solid melting fractions of $f_{s}=0.999$, according to the solutions from each model discussed in this work and obtained through the RHBIM.

The first term on this equation is exactly the volume expansion used in [18], as expressed through Equation (23). The second term represents the contribution to ΔV from the change of density experienced by the liquid phase. On the one hand, it is expected that for negligible density changes, both models show a good agreement for ${\tilde{k}}_{s}\ll \;B_{\ell }$, as illustrated in Figure 2. On the other hand, for moderate and high values of ${\tilde{k}}_{s}$, the contribution to ΔV from the change of density $\Delta \rho_{\ell }$ is not negligible. Within this regime, it is expected that the volume change according to Equation (23), leads to solutions with significant deviations from mass conservation. The mass of the PCM was obtained for $f_{s}=0.999$ and every value of ${\tilde{k}}_{s}$ considered in Figure 2 and Figure 3. According to the model proposed in [18], additional mass is created at moderate and large values of ${\tilde{k}}_{s}$ as observed in Figure 4. The total mass values shown in Figure 4 were calculated through the RHBIM by solving the model proposed in [18] and shown in red squares, and the proposed model in this work, whose solutions are shown in blue squares. Each symbol corresponds to the total mass of the PCM at melting fractions of $f_{s}=0.999$ and for every value of the elastic constant ${\tilde{k}}_{s}$ considered in Figure 2 and Figure 3.

Finally, the isochoric limit established through Equation (24) has been used to obtain the maximum pressure increment $\Delta P_{\max}$ where the approximation to the liquid–solid saturation line is valid. According to Equation (24), if the initial radius of the solid sphere $\bar{r}\left(0\right)$ is equal to R(0) (the PCM is initially in its solid phase), $\rho_{\ell }^{*}=\rho_{s}\left(0\right)$ in the isochoric limit. This establishes an upper bound for $\rho_{\ell }$ at full melting. On the one hand, at moderate values of ${\tilde{k}}_{s}=3.5\;B_{\ell }$, the semi-analytical solution to the model introduced in [18] for $\rho_{\ell }$ at melted solid fractions of $f_{s}=0.999$ is $\rho_{\ell }=2205.77\;\mathrm{kg}/m^{3}$, where the corresponding pressure is $P_{\max}=267.67\;\mathrm{MPa}$. This value is above the established boundary of $\rho_{\ell }^{*}=2192.00\;\mathrm{kg}/m^{3}$. On the other hand, the solution for ${\tilde{k}}_{s}=3.0\;B_{\ell }$ at $f_{s}=0.999$ is $\rho_{\ell }=2189.75\;\mathrm{kg}/m^{3}$, which lies below $\rho_{\ell }^{*}=\rho_{s}\left(0\right)$. The corresponding pressure at this value of ${\tilde{k}}_{s}$ is $P_{\max}=230.27\;\mathrm{MPa}$. This criterion was used to establish a maximum value of ${\tilde{k}}_{s}$, above which the thermo-mechanical model introduced in [18] has no applicability.

From the above discussion it follows that for the type of PCM studied in this work, we cannot consider pressure increments above $\Delta P_{\max}=230.1687\;\mathrm{MPa}$. Additionally, at these pressure increments, the first-order terms in ΔP that appear in the approximation for the liquid–solid saturation temperature given by Equation (10) still dominate over the second order terms. Then, we will assume that the approximation given by Equation (10) is still valid for $\Delta P_{\max}=230.1687\;\mathrm{MPa}$. According to the numerical and semi-analytical results shown in Figure 2, the proposed model in this work is well behaved within the pressure regime where the approximation to the liquid–solid saturation line is valid. Even at high values of ${\tilde{k}}_{s}=1000\;B_{\ell }$, the numerical and semi-analytical solutions to our model, approach asymptotically to the isochoric limit given by Equations (24) and (25).

### 2.6. Numerical and Semi-Analytical Results: Absorbed Thermal Energy

In this section, the results obtained for the sensible and latent heat absorbed by the PCM will be presented. The thermal energy absorbed during the melting process ΔQ is obtained through the stages previously discussed. For large values of ${\tilde{k}}_{s}$, the pressure increments obtained through the model presented in [18] lie outside the pressure domain, where the approximation given by Equation (10) is valid. Therefore, to compare the results of this work, with the model introduced in [18], only weak and moderate values of the spring constant will be considered.

**Figure 5:**
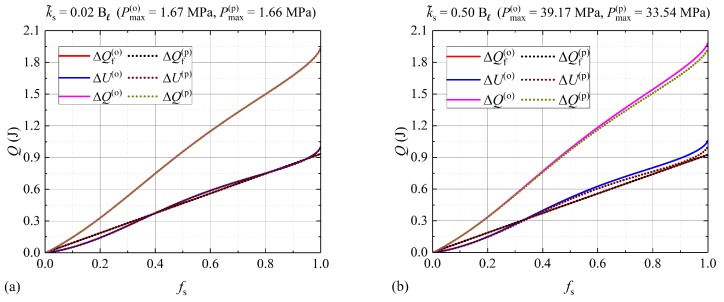
Absorbed energy for: (**a**) ${\tilde{k}}_{s}=0.02\;B_{\ell }$ and (**b**) ${\tilde{k}}_{s}=0.5\;B_{\ell }$. Sensible heat (ΔU), latent heat ($\Delta Q_{f}$), and total energy (ΔQ) absorbed by the nitrate salt during a melting process are shown for each value of ${\tilde{k}}_{s}$. Continuous and dotted lines correspond to the FDM solutions obtained from the model introduced in [18] and the proposed model, respectively.

In Figure 5, the sensible heat, latent heat, and total thermal energy absorbed by the PCM is shown for small values of ${\tilde{k}}_{s}$ where the transition is within the isobaric regime. The solutions obtained through the numerical and semi-analytical methods applied to both models are used to estimate the total energy according to Equations (14), (16), (17) and (19)–(21). The index (o) and (p) corresponds to the model proposed by other authors and the model proposed in this paper, respectively. The maximum pressure according to the model used by other authors $P_{\max}^{\left(o\right)}$ and the proposed model $P_{\max}^{\left(p\right)}$ for $f_{s}=0.999$ is also shown. In this figure, only the FDM solutions to the thermal energy absorbed by the PCM are shown. In all cases illustrated in Figure 5, the absorbed energy is obtained as a function of $f_{s}$, within a range of melted solid mass fraction, $f_{s}=\left[0,0.999\right]$.

**Figure 6:**
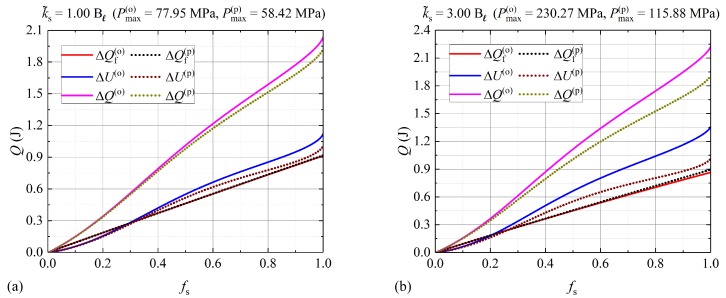
(**a**) ${\tilde{k}}_{s}=1.00\;B_{\ell }$ and (**b**) ${\tilde{k}}_{s}=3.00\;B_{\ell }$. Sensible heat (ΔU), latent heat ($\Delta Q_{f}$), and total energy (ΔQ) absorbed by the nitrate salt for each value of ${\tilde{k}}_{s}$ shown in this figure.

As illustrated in Figure 2, the model presented in [18] estimates larger pressure increments for moderate values of ${\tilde{k}}_{s}$, when compared to the proposed model. According to this result, it is expected that the latent heat of fusion drops faster at these values of ${\tilde{k}}_{s}$ than the thermo-mechanical model proposed in this work. This behavior is observed in Figure 3. Therefore, the solution to the model introduced in [18] is expected to underestimate the latent heat absorbed by the nitrate salt for moderate values of ${\tilde{k}}_{s}$, when compared to the proposed model. This effect is more evident in Figure 6, and for ${\tilde{k}}_{s}=3\;B_{\ell }$, where the FDM solution to $\Delta Q_{f}^{o}$ according to the model proposed in [18] (plotted as a continuous red line) lies slightly below the FDM solution to $\Delta Q_{f}^{p}$ obtained from the proposed model (plotted as a black dotted line). Even though, the relative percent difference (RPD) between the inner pressures predicted by each model for $f_{s}=0.999$ and ${\tilde{k}}_{s}=3\;B_{\ell }$ is practically 100%, the corresponding RPD in the latent heat absorbed is 3.36%. Then, for this type of PCM, the difference between in $\Delta Q_{f}$ between both models may not be significant.

Although there is a small difference in the predictions for the latent heat absorbed by the PCM, large differences are observed in the sensible heat absorbed, as illustrated in Figure 6. Additionally, large differences in the maximum pressures are observed, since the phase transition for the values of ${\tilde{k}}_{s}$ considered in this figure is well outside the isobaric regime. The difference in ΔU between both models is related to mass conservation. In Figure 4, it is shown that the solution to the model proposed in [18] tends to create mass. This effect is more evident for higher values of ${\tilde{k}}_{s}$. Then, compared to the model proposed in this work, more energy is stored as sensible heat according to the model introduced in [18]. In this case, to produce a given increment in the temperature of the PCM requires more energy, because there is more mass to store sensible heat. The expected behavior is shown in Figure 6, where the absorbed sensible heat according to the model proposed in [18] (plotted as a continuous blue line) overestimates the absorbed sensible heat obtained with the solutions of the model proposed in this work (plotted with a dotted brown line).

In Table 2 and Table 3, the RPD for ΔQ is shown. The RPD was obtained from the numerical and semi-analytical solutions for the total energy absorbed, and it is defined as:

(27) $$\mathrm{RPD}=\frac{\Delta Q^{\left(o\right)}-\Delta Q^{\left(p\right)}}{\Delta Q^{\left(p\right)}}\;\times \;100\%,$$

where $\Delta Q^{\left(o\right)}$ corresponds to the thermal energy absorbed according to other authors [18,19,20], and $\Delta Q^{\left(p\right)}$ is the corresponding solution for the thermal energy according to the model proposed in this work. The RPD and the values for the absorbed energy at different melted solid fractions are shown in Table 2 and Table 3. For small values of the spring constant ${\tilde{k}}_{s}$, the solid melts within the isobaric regime as illustrated in Figure 5. Within this domain of pressures, we expect to observe small values of the RPD, as shown in Table 2.

Table 3 shows the RPD in the energy absorbed, which is illustrated in Figure 6. The RPD between the two models according to the RHBIM and the FDM is calculated at several melted solid fractions $f_{s}$. According to the results illustrated in Figure 2 and Figure 3, significant differences between $\Delta Q^{\left(o\right)}$ and $\Delta Q^{\left(p\right)}$ are expected in this pressure domain.

## 3. Numerical and Semi-Analytical Methods

The proposed model described by Equations (4), (6), (7) and (9)–(12) is solved through a second order FDM and the semi-analytical RHBIM. Also, the thermal balance at the interface and volume expansion given by Equations (22) and (23), and Equations (9)–(11) and (13) which represent the model proposed in [18,19,20] are solved through the numerical and semi-analytical methods. All these methods were implemented in our own Fortran codes and Maple for mathematical manipulation of long expressions. Figure 1 was created with the Inkskape drawing software and graphs shown in Figure 2, Figure 3, Figure 4, Figure 5 and Figure 6, were produced by using the OriginLab software.

The initial temperature profile considered is a second order polynomial in *r* that satisfies the boundary conditions given by Equation (1), and the following initial conditions

(28) $$T_{s}\left(0,0\right)=T_{C},\;\;T_{\ell }\left(R\left(0\right),0\right)=T_{H},\;\;\frac{\partial \;T_{s}\left(r,0\right)}{\partial \;r}{|}_{r=0}=0\;\;\mathrm{and}\;\;\frac{\partial \;T_{\ell }\left(r,0\right)}{\partial \;r}{|}_{r=R(0)}=0.$$

The initial temperature distribution in the liquid and solid phase is given by the following second order polynomials

(29) $$\begin{array}{ccc} T_{s}\left(r,0\right) & = & a_{s}\left(0\right)\;\left(r-\bar{r}\left(0\right)\right)+b_{s}\left(0\right)\;{\left(r-\bar{r}\left(0\right)\right)}^{2}+T_{f}\left(0\right),\;\;\;\mathrm{with}\;\;\;0\leq \;r\;\leq \;\bar{r}\left(0\right)\;\;\;\mathrm{and}\\ T_{\ell }\left(r,0\right) & = & a_{\ell }\left(0\right)\;\left(r-\bar{r}\left(0\right)\right)+b_{\ell }\left(0\right)\;{\left(r-\bar{r}\left(0\right)\right)}^{2}+T_{f}\left(0\right),\;\;\;\mathrm{with}\;\;\;\bar{r}\left(0\right)\leq \;r\;\leq \;R\left(0\right). \end{array}$$

Here, the coefficients at phase i=ℓ(s) liquid(solid), $a_{i}$ and $b_{i}$ can be determined by applying the boundary conditions and initial conditions given by Equations (1) and (28), respectively. The fusion temperature $T_{f}\left(0\right)$, corresponds to the value of the liquid–solid saturation temperature at 1atm of pressure.

After performing the time discretization in both methods, Equations (4), (6), (7) and (9) constitute a set of nonlinear equations for the dynamical variables of motion $\bar{r}\left(t\right)$ and R(t), and the densities of each phase. The time discretized EMB equation at the interface is then, given by:

(30) $$\begin{array}{ccc} {}^{n}L_{f}\;\left[{}^{n}\rho_{\ell }\left(\frac{{}^{n}R^{2}}{{}^{n}{\bar{r}}^{2}}\frac{{}^{n+1}R-{}^{n}R}{\Delta t}-\frac{{}^{n+1}\bar{r}-{}^{n}\bar{r}}{\Delta t}\right)+\frac{1}{3}\;\frac{{}^{n+1}\rho_{\ell }-{}^{n}\rho_{\ell }}{\Delta t}\left(\frac{{}^{n}R^{2}}{{}^{n}{\bar{r}}^{2}}{}^{n}R-{}^{n}\bar{r}\right)\right] & = & \dot{q}\left({}^{n}\bar{r}\right), \end{array}$$

where ${}^{n}\bar{r}$$\left({}^{n+1}\bar{r}\right)$, ${}^{n}R$$\left({}^{n+1}R\right)$ and ${}^{n}\rho_{\ell }$$\left({}^{n+1}\rho_{\ell }\right)$ are the interface position, radius of the sphere, and liquid density at the *n*th(*n*th + 1) time level. Also, in Equation (30), the net heat flux per unit area at the interface has been defined as

(31) $$\dot{q}\left({}^{n}\bar{r}\right)=\lambda_{\ell }\frac{\partial \;T_{\ell }\left(r,t\right)}{\partial \;r}{|}_{r=\bar{r}\left(t\right)}-\lambda_{s}\frac{\partial \;T_{s}\left(r,t\right)}{\partial \;r}{|}_{r=\bar{r}\left(t\right)}.$$

The heat flux $\dot{q}\left({}^{n}\bar{r}\right)$ is obtained by using a fourth order approximation in Δr to the spatial derivative when using the FDM [26]. The semi-analytical approach is to estimate $\dot{q}\left({}^{n}\bar{r}\right)$, through continuous temperature distributions [26]. The equation for mass conservation (6) is discretized as follows:

(32) $$\begin{array}{ccc} \frac{dM_{\mathrm{PCM}}\left(t\right)}{dt} & = & {}^{n}{\bar{r}}^{2}[{}^{n}\rho_{s}\frac{{}^{n+1}\bar{r}-{}^{n}\bar{r}}{\Delta t}+\frac{1}{3}\frac{{}^{n+1}\rho_{s}-{}^{n}\rho_{s}}{\Delta t}\;\;{}^{n}\bar{r}+{}^{n}\rho_{\ell }\left(\frac{{}^{n}R^{2}}{{}^{n}{\bar{r}}^{2}}\;\frac{{}^{n+1}R-{}^{n}R}{\Delta t}-\frac{{}^{n+1}\bar{r}-{}^{n}\bar{r}}{\Delta t}\right)\\ & & +\frac{1}{3}\;\frac{{}^{n+1}\rho_{\ell }-{}^{n}\rho_{\ell }}{\Delta t}\left(\frac{{}^{n}R^{2}}{{}^{n}{\bar{r}}^{2}}\;{}^{n}R-{}^{n}\bar{r}\right)]=0, \end{array}$$

where ${}^{n}\rho_{s}$$\left({}^{n+1}\rho_{s}\right)$ is the density of the solid phase at the *n*th(*n*th + 1) time level. Finally, the deformation of each phase (7), and the coupling between the liquid and spring array deformation (9) are discretized as:

(33) $$B_{\ell }\left(\frac{{}^{n+1}\rho_{\ell }-{}^{n}\rho_{\ell }}{{}^{n+1}\rho_{\ell }}\right)=B_{s}\left(\frac{{}^{n+1}\rho_{s}-{}^{n}\rho_{s}}{{}^{n+1}\rho_{s}}\right),$$

and

(34) $$B_{\ell }\left(\frac{{}^{n+1}\rho_{\ell }-{}^{n}\rho_{\ell }}{{}^{n+1}\rho_{\ell }}\right)={\tilde{k}}_{s}\frac{{}^{n+1}R-{}^{n}R}{R_{0}}.$$

The same discretization is used to solve the model introduced in [18]. In this case, the dynamic variables and the density of the liquid are uncoupled, since density changes are neglected in the thermal balance at the interface and in the estimation of ΔV. Then, the interface position can be solved directly from Equation (22) as follows

(35) $${}^{n+1}\bar{r}={}^{n}\bar{r}+\frac{\dot{q}\left({}^{n}\bar{r}\right)}{{}^{n}L_{f}\;\rho_{s}}.$$

From the volume change given by Equation (23), the radius of the PCM is given by:

(36) $${}^{n+1}R={\left[{}^{n}R^{3}+\left(\frac{\rho_{s}}{\rho_{\ell }\left(0\right)}-1\right)\left({}^{n}{\bar{r}}^{3}-{}^{n+1}{\bar{r}}^{3}\right)\right]}^{1/3},$$

where the interface position at the *n*th + 1 time level can be substituted from Equation (35). Equation (33) is not necessary, given that in this approximation the solid is assumed to be incompressible. Finally, the density of the liquid phase can be solved from Equation (34).

The solutions for ${}^{n}\bar{r}$, ${}^{n}R$, ${}^{n}\rho_{\ell }$ and ${}^{n}\rho_{s}$ are used to obtain the pressure increment ${}^{n}\Delta P={}^{n}P-P_{\mathrm{atm}}$ at the *n*th time level. Finally, the estimated pressure increment according to each model is used to obtain the fusion temperature ${}^{n}T_{f}$ and the latent heat of fusion ${}^{n}L_{f}$ from Equations (10) and (11), respectively.

### 3.1. FDM

The difference between the numerical and semi-analytical methods used in this work, lies in the way in which the temperature field is estimated. The spatial derivatives that appear in Equations (12) and (13) are approximated up to second order terms in Δr. An implicit finite difference scheme is used [26], with a backward difference definition for the time derivatives that appear in Equations (12) and (13). The liquid(solid) phase domain is discretized by using a mesh with a constant number of nodes $M_{1}+1$($M_{2}+1$). Additionally, the mesh is moved according to the dynamics of the interface and the system size [26]. A central definition for the first and second order spatial derivatives is used, so a system of $M_{1}\left(M_{2}\right)$ equations for the temperature ${}^{n+1,m}T_{\ell }$(${}^{n+1,m}T_{s}$) at each node is obtained [27]. The mesh size for the FDM used in this work was chosen with 161(161) nodes in the liquid(solid) phase. For a solid sphere with an initial radius of $\bar{r}\left(0\right)=0.99\;\mathrm{mm}$ and a liquid shell with a thickness of 0.001mm, the initial spatial step Δr in the solid(liquid) is ${}^{0}\Delta r_{s}=6.19\;\mu$m (${}^{0}\Delta r_{\ell }=0.0063\;\mu$m). The time step used in the simulations was Δt=0.5μs.

### 3.2. RHBIM

The RHBIM used in this work has been fully described elsewhere [24,25,28,29] for one-dimensional problems in rectangular coordinates. However, a brief description of the methodology used in this work will be given. The solid sphere and liquid shell are divided in M and N regions, respectively. Continuous and smooth functions for the temperature distributions are proposed in each phase. The accuracy of the method depends on the type of temperature profile proposed, which classically is a quadratic polynomial [28,30]. However, depending on the nature of the boundary conditions and the geometry of the system, other profiles have been used [28,30]. In this work, parabolic profiles have been proposed within each region of the solid and liquid phase, as follows

(37) $$\begin{array}{ccc} {}^{m}T_{s}\left(r,t\right) & = & {}^{m}a_{s}\left(t\right)\;\left(r-{}^{m}r_{s}\left(t\right)\right)+{}^{m}b_{s}\left(t\right)\;{\left(r-{}^{m}r_{s}\left(t\right)\right)}^{2}+{}^{m}T_{(}t),\;\;\;\mathrm{with}\;\;\;1\leq \;m\;\leq \;M\;\;\;\mathrm{and}\\ {}^{n}T_{\ell }\left(r,t\right) & = & {}^{n}a_{\ell }\left(t\right)\;\left(r-{}^{n}r_{\ell }\left(t\right)\right)+{}^{n}b_{\ell }\left(t\right)\;{\left(r-{}^{n}r_{\ell }\left(t\right)\right)}^{2}+{}^{n}T\left(t\right),\;\;\;\mathrm{with}\;\;\;1\leq \;n\;\leq \;N. \end{array}$$

Here the time dependent coefficients ${}^{m}a_{s}\left(t\right)$ and ${}^{n}a_{\ell }\left(t\right)$ within each region of the solid and liquid phases, and the temperature values ${}^{m}T\left(t\right)$ and ${}^{n}T\left(t\right)$ for each of the solid and liquid regions, can be expressed in terms of the coefficients ${}^{m}b_{s}\left(t\right)$ and ${}^{n}b_{\ell }\left(t\right)$. This can be done through the boundary conditions given by Equation (1) and by imposing continuity and smoothness at each boundary between adjacent regions, ${}^{m}r_{s}\left(t\right)$ and ${}^{n}r_{\ell }\left(t\right)$ [29]. These boundaries are coupled to the dynamical behavior of the interface and radius of the PCM [24,25]; therefore, the time dependence of the boundaries between adjacent regions is given by:

(38) $${}^{m}r_{s}\left(t\right)=\bar{r}\left(t\right)\frac{m}{M}$$

for the solid sphere, and

(39) $${}^{m}r_{\ell }\left(t\right)=\bar{r}\left(t\right)+\frac{n}{N}\left(R\left(t\right)-\bar{r}\left(t\right)\right),$$

for the liquid shell.

The temperature distributions given by Equation (37) are used to average Equations (12) and (13) over the spatial variable, as described in [25]. After the averaging process, a system of M(N) ordinary differential equations (ODEs) in time is found for the time dependent coefficients, ${}^{m}b_{s}\left(t\right)$(${}^{n}b_{\ell }\left(t\right)$). The resulting system of ODEs is discretized by using a forward difference approximation for the time derivative. Finally, each coefficient can be found for the next time level by solving the resulting system of algebraic equations. In this work, Equations (12) and (13) were averaged over 3 regions in each phase, resulting in 3 ODEs for ${}^{m}b_{s}\left(t\right)$ and 3 ODEs for ${}^{n}b_{\ell }\left(t\right)$. The nonlinear system of equations described at the beginning of this section, and the linear system of equations for the time dependent coefficients ${}^{m}b_{s}\left(t\right)$ and ${}^{n}b_{\ell }\left(t\right)$, were solved by using a time step of Δt=0.5μs until the system reached melted solid fractions of $f_{s}=0.999$.

## 4. Conclusions

Several results can be outlined from this work. From the theoretical and physical points of view, mass conservation constitutes the central idea for estimating the contribution of density changes to the absorbed energy. Total mass conservation leads to an EMB equation at the interface, which for a spherical configuration is found to have an extra term that depends on the geometry of the system and rate of density changes. This extra term represents a small perturbation, when the melting process takes place near the isobaric regime. Within this pressure domain, the proposed solutions in this work, show a good agreement with the solutions from other authors. At high pressures, the contributions from density changes in the EMB at the interface and mass conservation equation are significant. Large differences between the proposed solutions to the densities and liquid–solid saturation line, and the predictions from other authors are observed in this pressure domain. Isochoric limits for the density, pressure, and liquid–solid saturation point, were obtained by assuming that total mass is also conserved in this regime. The solutions obtained through the proposed model are observed to be well behaved near the isochoric regime, and found to approach asymptotically to the predicted limit.

The most important finding in this work, corresponds to the effects of density changes in the thermal energy absorbed by the phase-change material. The sensible heat absorbed during the melting process was conceived through total and local mass conservation. According to the numerical and semi-analytical solutions to the model proposed in this work, the energy absorbed by the nitrate salt can be significantly overestimated by the solutions from other authors. On the one hand, the sensible and latent heat absorbed near the isobaric regime, show a good agreement between both models. On the other hand, at large pressures, where the approximation used for the liquid–solid saturation line is still valid, significant differences in the absorbed energy were observed. Within this pressure domain, and according to the numerical and semi-analytical solutions, an approximate difference of 16% between the predictions from both models, can be expected. This difference in the estimation of the heat storage capacity of TES units has a direct impact on the design and efficient use of renewable energy in CSP plants and domestic house heating applications.

Finally, experimental validation of these results is still missing. However, by using materials with low thermal expansion coefficients or keeping a small temperature range, the solutions to the proposed model are expected to be well behaved at high pressures when compared to experimental results. We have still to elaborate a model which is capable of taking into account thermal expansion effects on encapsulated PCMs. These effects may also have an impact on the thermal energy absorbed by the system. However, the EMB equation at the interface will be completely different when including these effects. Additionally, the role of mass conservation for obtaining the liquid and solid deformation during the melting process when the densities depend on the temperature profile is still not clear. 

## Figures and Tables

**Table 1:** Thermodynamic data for the nitrite salt ${\mathrm{KNO}}_{3}/{\mathrm{NaNO}}_{3}$ at 1atm [18].

State	Property	Salt
Liquid	$\rho_{\ell }\left(0\right)$ ($\mathrm{kg}/m^{3}$)	2096.0
	$C_{\ell }$ (kJ/kg·K)	1.500
	$k_{\ell }$ (W/m·K)	0.80
	$\alpha_{\ell }$ (1/K)	$3.7\times 10^{-4}$
	$B_{\ell }$ (Pa)	$5.38\times 10^{9}$
Solid	$\rho_{s}\left(0\right)$ ($\mathrm{kg}/m^{3}$)	2192.0
	$C_{s}$ (kJ/kg·K)	1.430
	$k_{s}$ (W/m·K)	1.0
	$\alpha_{s}$ (1/K)	0
	$B_{s}$ (Pa)	$5.38\times 10^{15}$
Liquid–Solid	$T_{f}\left(0\right)$ (K)	496.15
	$L_{f}\left(0\right)$ (kJ/kg)	105.0

**Table 2:** Calculated values of ΔQ at different melted solid fractions $f_{s}$.

${\tilde{k}}_{s}=0.02\;B_{\ell }$						
	**RHBIM**			**FDM**		
**$f_{s}$**	$\Delta Q^{\left(o\right)},\left(J\right)$	$\Delta Q^{\left(p\right)}\;\left(J\right)$	RPD%	$\Delta Q^{\left(o\right)}\;\left(J\right)$	$\Delta Q^{\left(p\right)}\;\left(J\right)$	RPD%
0.200	0.322851	0.322751	0.031009	0.329669	0.329570	0.030014
0.400	0.750799	0.750426	0.049719	0.749478	0.749107	0.049590
0.600	1.151147	1.150360	0.068445	1.149879	1.149091	0.068530
0.800	1.500673	1.499320	0.090199	1.500158	1.498806	0.090224
0.999	1.952634	1.950478	0.110516	1.931832	1.929690	0.110999
${\tilde{k}}_{s}=0.50\;B_{\ell }$						
	**RHBIM**			**FDM**		
**$f_{s}$**	$\Delta Q^{\left(o\right)}\;\left(J\right)$	$\Delta Q^{\left(p\right)},\left(J\right)$	RPD%	$\Delta Q^{\left(o\right)}\;\left(J\right)$	$\Delta Q^{\left(p\right)}\;\left(J\right)$	RPD%
0.200	0.328046	0.325425	0.805233	0.335435	0.332826	0.784106
0.400	0.771019	0.760911	1.328459	0.769567	0.759496	1.325984
0.600	1.184663	1.163518	1.817271	1.183411	1.162257	1.820062
0.800	1.544538	1.509094	2.348696	1.544044	1.508598	2.349626
0.999	1.993065	1.944339	2.506055	1.981092	1.927143	2.799454

**Table 3:** Calculated values of ΔQ for ${\tilde{k}}_{s}=B_{\ell }$ and ${\tilde{k}}_{s}=3\;B_{\ell }$.

${\tilde{k}}_{s}=\;B_{\ell }$						
	**RHBIM**			**FDM**		
**$f_{s}$**	$\Delta Q^{\left(o\right)},\left(J\right)$	$\Delta Q^{\left(p\right)}\;\left(J\right)$	RPD%	$\Delta Q^{\left(o\right)}\;\left(J\right)$	$\Delta Q^{\left(p\right)}\;\left(J\right)$	RPD%
0.200	0.333506	0.328038	1.667047	0.341453	0.335974	1.630623
0.400	0.791873	0.770294	2.801517	0.790260	0.768759	2.796869
0.600	1.218525	1.174065	3.786892	1.217289	1.172801	3.793311
0.800	1.588553	1.515688	4.807451	1.588082	1.515206	4.809648
0.999	2.050789	1.943153	5.539260	2.031265	1.923613	5.596347
${\tilde{k}}_{s}=3.0\;B_{\ell }$						
	**RHBIM**			**FDM**		
**$f_{s}$**	$\Delta Q^{\left(o\right)}\;\left(J\right)$	$\Delta Q^{\left(p\right)},\left(J\right)$	RPD%	$\Delta Q^{\left(o\right)}\;\left(J\right)$	$\Delta Q^{\left(p\right)}\;\left(J\right)$	RPD%
0.200	0.355787	0.336985	5.579661	0.365541	0.346518	5.489847
0.400	0.871734	0.796825	9.400960	0.869372	0.794792	9.383609
0.600	1.340701	1.198014	11.910236	1.339509	1.196714	11.932198
0.800	1.744361	1.525478	14.348484	1.743959	1.525026	14.356043
0.999	2.235443	1.927275	15.989813	2.218157	1.908944	16.198151

## Abbreviations

The following abbreviations and symbols are used in this manuscript:

PCM		phase change material
HTPCM		high-temperature phase-change material
TES		thermal energy storage
CSP		concentrating solar power plant
HTF		heat-transfer fluid
EMB		energy-mass balance
FDM		finite difference method
RHBIM		refined heat balance integral method
RPD		relative percent difference
$\lambda_{\ell }$	W/mK	Thermal conductivity of the liquid
$\lambda_{s}$	W/mK	Thermal conductivity of the solid
$\alpha_{\ell }$	1/K	Thermal expansion coefficient of the liquid
$\alpha_{s}$	1/K	Thermal expansion coefficient of the solid
$C_{\ell }$	kJ/kgK	Specific heat capacity of the liquid
$C_{s}$	kJ/kgK	Specific heat capacity of the solid
$B_{\ell }$	Pa	Bulk modulus of the liquid
$B_{s}$	Pa	Bulk modulus of the solid
$K_{\ell }$	1/Pa	Compressibility of the liquid
$K_{s}$	1/Pa	Compressibility of the solid
$\rho_{\ell }\left(t\right)$	$\mathrm{kg}/m^{3}$	Time dependent liquid density
$\rho_{s}\left(t\right)$	$\mathrm{kg}/m^{3}$	Time dependent solid density
$\rho_{\ell }^{*}$	$\mathrm{kg}/m^{3}$	Density of the liquid in the isochoric limit
$P^{*}$	MPa	Inner pressure in the isochoric limit
$T_{f}^{*}$	K	Fusion temperature in the isochoric limit
$L_{f}^{*}$	kJ/kg	Latent heat of fusion in the isochoric limit
$T_{f}\left(t\right)$	K	Time dependent fusion temperature
$L_{f}\left(t\right)$	kJ/kg	Time dependent latent heat of fusion
R(t)	m	Time dependent PCM radius
$\bar{r}\left(t\right)$	m	Time dependent radius of the solid phase
$T_{H}$	K	Temperature at the surface of the PCM
${\tilde{k}}_{s}$	Pa	Elastic constant of the spring
ΔU	J	Internal energy change
ΔV	$m^{3}$	Total volume change
$\Delta Q_{f}$	J	Energy absorbed as latent heat
ΔQ	J	Thermal energy absorbed
$M_{\mathrm{PCM}}$	mg	Total mass of the PCM
$\Delta M_{s}$	mg	Mass of melted solid
$t_{\max}$	s	Duration of the melting process
